# Tenodese do trato iliotibial no tratamento das lesões do complexo anterolateral do joelho – Descrição de uma técnica modificada

**DOI:** 10.1055/s-0045-1811631

**Published:** 2025-11-10

**Authors:** Gustavo Rabelo Azi, Alexandre Vasconcelos de Meirelles, Ramon Rocha Costa de Freitas, José Fonseca Fróes Neto, Enilton de Santana Ribeiro de Mattos, Alex Guedes

**Affiliations:** 1 Grupo de Cirurgia do Joelho, Hospital Santo Antônio, Obras Sociais Irmã Dulce, Salvador, BA, Brasil; 2 Programa de Residência Médica em Ortopedia e Traumatologia, Hospital Santo Antônio, Obras Sociais Irmã Dulce, Salvador, BA, Brasil; 3 Grupo de Cirurgia do Quadril, Hospital Santo Antônio, Obras Sociais Irmã Dulce, Salvador, BA, Brasil; 4 Grupo de Cirurgia da Mão, Hospital Santo Antônio, Obras Sociais Irmã Dulce, Salvador, BA, Brasil; 5 Grupo de Cirurgia do Joelho, Hospital Universitário Professor Edgard Santos, Faculdade de Medicina da Bahia, Universidade Federal da Bahia, Salvador, BA, Brasil

**Keywords:** fáscia lata, instabilidade articular, joelho, ligamentos, procedimentos cirúrgicos operatórios, reconstrução do ligamento cruzado anterior, anterior cruciate ligament reconstruction, fascia lata, joint instability, knee, ligaments, surgical procedures operative

## Abstract

Os autores descrevem uma técnica modificada de tenodese extrarticular lateral para o tratamento das lesões do complexo anterolateral do joelho, realizada em associação à reconstrução do ligamento cruzado anterior, utilizando enxerto tendíneo quádruplo de semitendíneo e grácil. A fixação femoral do enxerto é realizada com um único parafuso de interferência, de fora para dentro, compartilhando o mesmo túnel ósseo com a banda central do trato iliotibial. A entrada do túnel é posicionada na topografia do epicôndilo lateral, ao nível da origem do ligamento anterolateral. O protocolo pós-operatório inclui fisioterapia nos primeiros 4 meses, seguida pelo início do reforço muscular, com liberação para a prática esportiva a partir do 9° mês.

## Abstract (en)

The authors describe a modified lateral extraarticular tenodesis technique for the treatment of anterolateral complex knee injuries, performed in association with anterior cruciate ligament reconstruction, using a quadruple semitendinosus and gracilis tendon graft. Femoral fixation of the graft is performed with a single interference screw, together with the central band of the iliotibial tract, from the outside in, sharing the same bone tunnel, whose entrance is positioned in the topography of the lateral epicondyle (at the level of the origin of the anterolateral ligament). The postoperative protocol includes physiotherapy during the first 4 months, followed by the initiation of muscle strengthening, with clearance for return to sports starting from the 9 ^th^ month.

## Introdução

A instabilidade rotatória anterolateral (IRA) decorre da ruptura do ligamento cruzado anterior (LCA) associada à lesão do complexo anterolateral do joelho (porção do trato iliotibial [TIT] situada entre as fibras de Kaplan proximalmente e a sua inserção tibial), 1 2 gerando movimento anômalo translacional anterior e rotacional (interno) da tíbia. 2

A falha em reconhecer e manejar lesões concomitantes do complexo anterolateral do joelho no momento da reconstrução do LCA pode resultar em IRA persistente, culminando em pior resultado funcional, falha da reconstrução/ruptura do neoligamento e osteoartrite progressiva. 2 3 Diante disso, a abordagem cirúrgica concomitante do complexo anterolateral, incluindo o ligamento anterolateral (LAL) e o TIT, tornou-se objeto de crescente interesse e investigação, por permitir o restabelecimento da estabilidade rotacional, restaurando a biomecânica normal do joelho, reduzindo as taxas de falha da reconstrução isolada do ligamento e prevenindo ou minimizando outras complicações. 2 3 4 5 6

Atualmente, duas técnicas são utilizadas no tratamento da instabilidade anterolateral: a tenodese extrarticular lateral (TEL) 3 7 8 9 e a reconstrução 2 4 do TIT.

A TEL 3 7 8 9 envolve a passagem do TIT através de túnel no fêmur, seguida por sua fixação a este osso, proporcionando contenção rotacional e estabilização lateral do joelho.

O objetivo do presente trabalho é descrever uma técnica de TEL modificada para o tratamento concomitante das lesões combinadas do LCA e do complexo anterolateral do joelho nas reconstruções em que é utilizado enxerto tendíneo quádruplo de semitendíneo e grácil. A fixação femoral do enxerto é realizada em conjunto com a banda central do TIT, compartilhando o mesmo túnel ósseo.

## Descrição da Técnica


**Vídeo 1**


O paciente é posicionado em decúbito dorsal e anestesiado mediante bloqueio raquidiano e sedação. A antibioticoterapia venosa profilática é completada até 30 minutos antes da incisão: utilizamos 1 g de cefazolina ou 600 mg de clindamicina (indicada em pacientes alérgicos a antibióticos betalactâmicos).

**Fig. 1:**
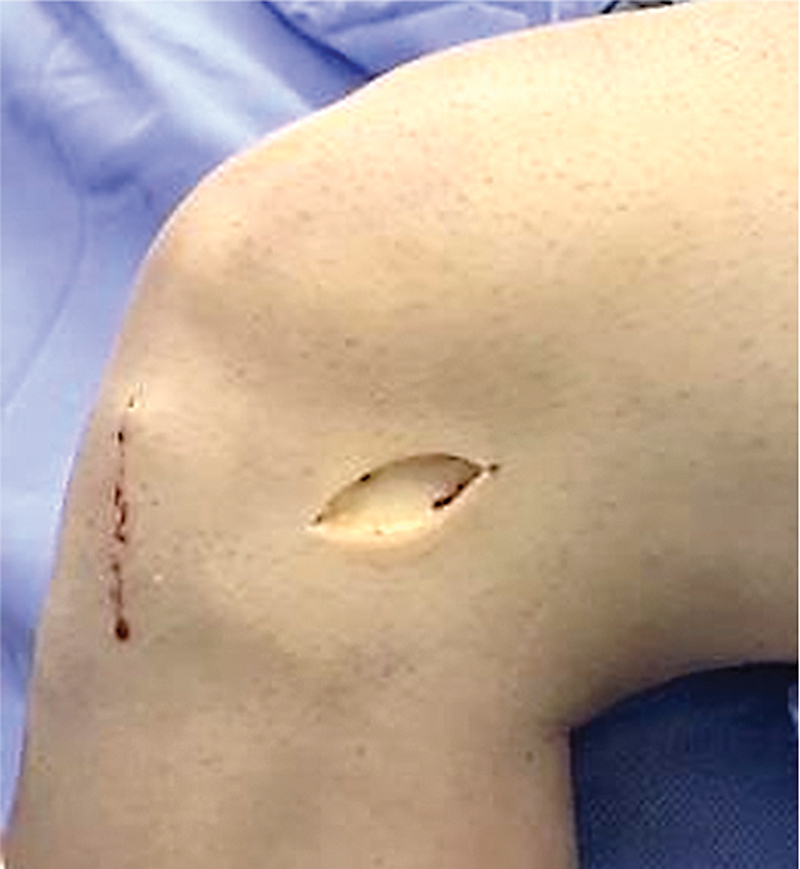
Mini acesso sobre o trato iliotibial.

Realizamos o esvaziamento venoso mediante elevação gravitacional do membro afetado por 5 minutos, seguido pelo uso de faixa de Esmarch e o garroteamento proximal, na raiz da coxa, com manguito pneumático. Com o joelho fletido a 90° e a perna pendente ao lado da mesa operatória, realizamos a abordagem rotineira para reconstrução anatômica do LCA, utilizando o enxerto tendíneo quádruplo de semitendíneo e grácil (confeccionado mediante sutura das extremidades e dobra na metade de seu comprimento), deixando o fio de sutura passado ao redor da dobra do enxerto, à exceção da confecção do túnel femoral, que é realizada de fora para dentro, com entrada posicionada na origem do LAL, 0,5 cm proximal e 0,5 cm posterior ao epicôndilo lateral do fêmur. Para tanto, realizamos mini acesso cutâneo (∼ 2,0 cm) na topografia do epicôndilo lateral, procedendo com a dissecção por planos, identificação e dissecção do TIT ( Fig. 1 ); uma vez dissecado, o TIT é incisado longitudinalmente (entre 5,0 e 7,5 cm), seguindo a orientação de suas fibras; o afastamento das bordas desta incisão permite identificar o epicôndilo lateral do fêmur ( Vídeo 1 ).

A seguir, posicionamos o fio guia ponta de broca 0,5 cm proximal e 0,5 cm posterior ao epicôndilo lateral do fêmur, direcionando-o ao centro do *footprint* do LCA, utilizando guia tipo *pinpoint* ; ato contínuo, confeccionamos túnel femoral de fora para dentro, no diâmetro calculado para a passagem do enxerto. O túnel tibial é confeccionado de fora para dentro; posicionamos o guia no *footprint* ou sobre o remanescente do ligamento cruzado anterior na tíbia para que a reconstrução fique anatômica ( Vídeo 1 ).

**Fig. 2:**
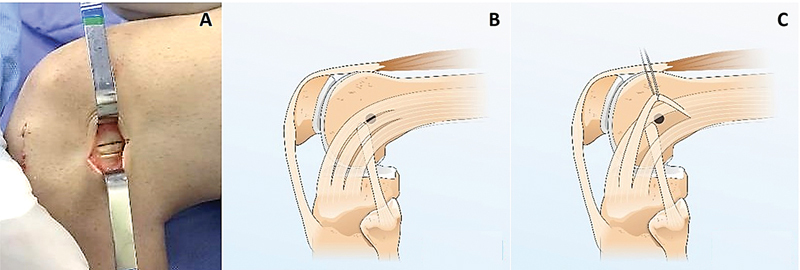
( **A-C** ). ( **A** ) Face lateral de um joelho esquerdo, onde se nota a incisão no trato iliotibial; ( **B** ) Ilustração do enxerto do trato iliotibial e o ponto de criação do túnel femoral; ( **C** ) enxerto do trato iliotibial sendo tracionado por um fio.

**Fig. 3:**
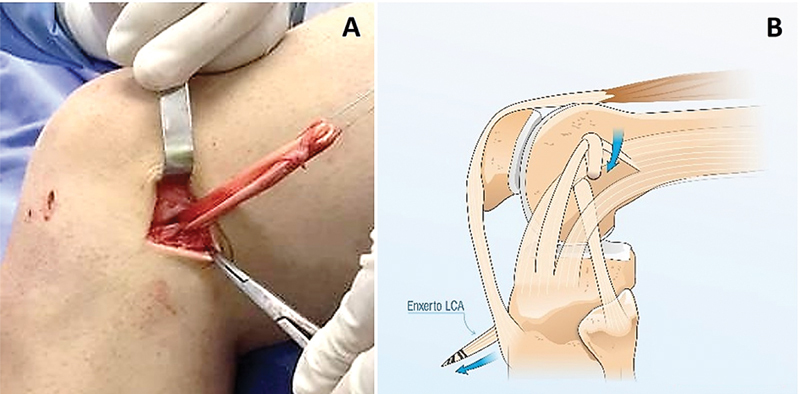
( **A-B** ). ( **A** ) Enxerto da banda central do TIT.; ( **B** ) Passado ao redor do enxerto dos flexores.

**Fig. 4:**
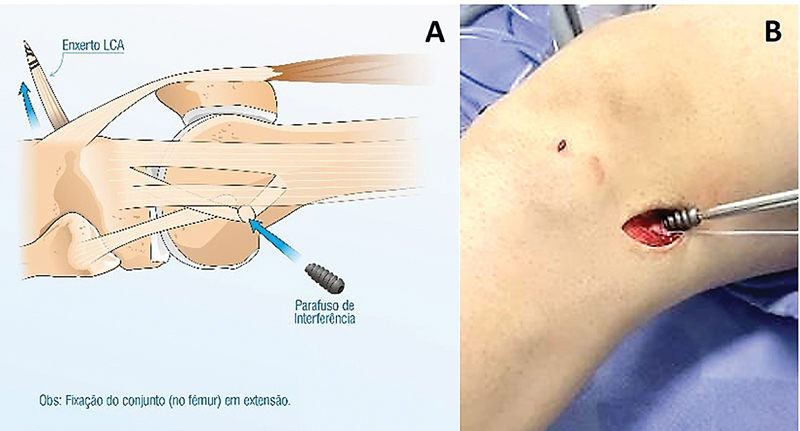
( **A-B** ). ( **A** ) Fixação da banda central do TIT, junto ao enxerto quádruplo de semitendíneo e grácil no túnel femoral, fixado com um parafuso e o joelho em extensão.

Realizamos nova incisão no TIT, paralela à inicial, distando 0,5 cm desta, criando uma banda central medindo 5,0–7,5 × 0,5 cm, mantendo continuidade com suas porções proximal e distal ( Fig. 2 ). Posicionamos o enxerto de proximal para distal, ao redor da banda ( Fig. 3 ), que é tracionada para a articulação mediante o fio de alta resistência (Ethibond 5, Johnson & Johnson) por alguns milímetros, no interior do túnel femoral; em seguida, com o joelho em rotação neutra e em extensão total, realizamos a fixação dessas estruturas com parafuso de interferência ( Fig. 4 , Vídeo 1 ).

**Fig. 5:**
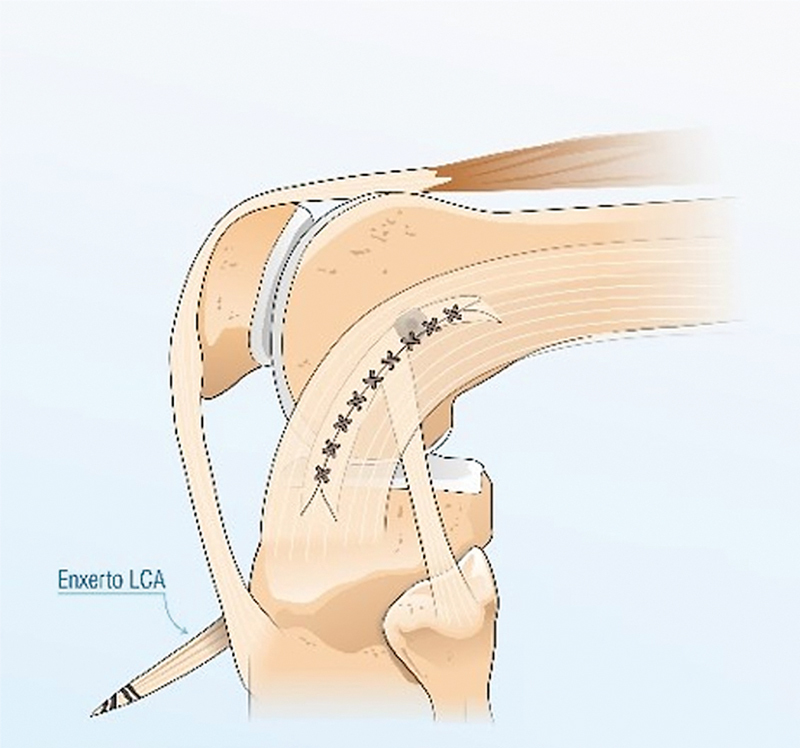
Sutura da banda central do TIT.

A seguir, com o joelho posicionado a 30° de flexão, realizamos a fixação do enxerto no túnel tibial, com parafuso de interferência. A falha criada pela invaginação da banda central do TIT no túnel femoral é então suturada com o joelho em flexão ( Fig. 5 , Vídeo 1 ) mediante pontos separados em “X”, utilizando fio Vycril 0 (Ethicon ou Johnson & Johnson Medical Devices). O procedimento é finalizado com a aproximação da ferida por planos, utilizando fio Vycril 2–0 (subcutâneo, pontos simples separados, nó invertido) e Mononylon 3–0 (Ethicon or Johnson & Johnson Medical Devices) (pele, pontos simples separados), curativo compressivo e exame radiográfico convencional de controle. Não utilizamos dreno aspirativo, tampouco imobilizador de joelho.

O paciente permanece internado por 1 dia, sendo liberada carga total já no 1° dia pós-operatório, conforme tolerada pelo paciente, utilizando muletas apenas para equilíbrio. A profilaxia de tromboembolismo venoso é realizada de forma mecânica exclusiva, mediante compressão mecânica intermitente e/ou uso de meias elásticas. É realizada crioterapia local e, conforme demanda, controle da dor mediante utilização de analgésicos não opioides. Os pontos são retirados com 15 dias.

O paciente é seguido mensalmente até o início das atividades desportivas, aos 9 meses de pós-operatório. A reabilitação pós-operatória segue o protocolo descrito a seguir:

- Do pós-operatório imediato até o terceiro mês: fisioterapia voltada para analgesia, ganho de amplitude articular e prevenção da perda de massa muscular.
- Entre o terceiro e o sexto mês de pós-operatório: transição gradual da fisioterapia para a prática de exercícios físicos, com foco no ganho progressivo de massa muscular.
- Entre o terceiro e o nono mês de pós-operatório: treinamento neuromuscular e de gestos esportivos, supervisionado por educador físico.
- Aos nove meses de pós-operatório: avaliação clínica para liberação à prática esportiva, incluindo análise da massa muscular, testes ligamentares, *hop test* e ressonância magnética de controle.

## Comentários Finais

A IRA decorre da ruptura do LCA associada à lesão do complexo anterolateral do joelho. A identificação desta condição demanda planejamento de abordagem cirúrgica concomitante, associando a reconstrução do LCA à TEL 3 7 8 9 ou à reconstrução 2 4 do TIT. As indicações primárias para este procedimento incluem re-ruptura do LCA, exame físico com *pivot shift* grau 2 ou 3, prática esportiva com movimentos de *pivot* e/ou prática esportiva de alto nível, frouxidão ligamentar e fratura de Segond; as indicações secundárias abrangem lesão crônica do LCA, idade < 25 anos e sinal radiográfico do entalhe do côndilo femoral lateral. 10

Uma das técnicas mais amplamente estudadas é a TEL, 3 7 8 9 apresentada por Marcel Lemaire, cirurgião francês, em 1967. 3 Há controvérsias sobre o papel preciso e a técnica ideal para a realização deste procedimento, levando várias propostas de modificações que emergiram como preferenciais, tendo em vista sua significativa eficácia na redução das taxas de falha da reconstrução isolada do LCA. 3 7 8 9

Apesar dos benefícios, é preciso considerar que a TEL apresenta algum potencial para complicações, 8 como dor, restrição da amplitude articular (sobretudo da flexão, devido ao tensionamento do TIT), falha na fixação e ruptura do enxerto.

Na TEL modificada, descrita no presente trabalho, o túnel femoral confeccionado para a passagem do enxerto tendíneo quádruplo do semitendíneo e grácil é compartilhado com a banda central do TIT, sendo ambos (enxerto e banda) fixados em conjunto, com um único parafuso de interferência. Nesta técnica, o ponto definido para confecção do túnel femoral (de fora para dentro, 0,5 cm proximal e 0,5 cm posterior ao epicôndilo lateral do fêmur, direcionado ao centro do *footprint* do LCA) considera a realização da tenodese no local mais próximo à origem do ligamento anterolateral, sem prejudicar o posicionamento do enxerto tendíneo quádruplo.

Dentre os benefícios deste procedimento, é possível enumerar: (1) único mini acesso – não há necessidade de nova incisão para a TEL; (2) procedimento tecnicamente mais simples; (3) menor tempo cirúrgico (menor tempo de garrote); (4) não é necessário modificar o protocolo de reabilitação pós-operatória da reconstrução do LCA em virtude da TEL; (5) menor demanda por implantes; e (6) menor custo, considerando os itens anteriores.

O potencial de risco para complicações é similar ao observado em outras técnicas de TEL, como (1) dor; (2) restrição da flexão; (3) falhas na fixação; e (4) ruptura do enxerto.
